# Cross-model evaluation of phishing detectors against LLM-generated emails

**DOI:** 10.3389/fdata.2026.1883452

**Published:** 2026-07-07

**Authors:** Rommel Gutierrez, William Villegas-Ch, Jaime Govea

**Affiliations:** Escuela de Ingeniería en Ciberseguridad, FICA, Universidad de las Américas, Quito, Ecuador

**Keywords:** AI-generated text, cross-model generalization, cybersecurity, large language models, machine learning, phishing detection, stylometric analysis, threshold calibration

## Abstract

Phishing remains a prevalent cyberattack vector, and the widespread adoption of large language models (LLMs) has enabled adversaries to generate grammatically correct and contextually coherent phishing emails at scale, against which conventional detection systems are less effective. Although stylometric methods achieve over 95% accuracy within a single generator, their performance has not been systematically evaluated when the source model changes between training and deployment. This represents a significant gap, as adversaries can switch generators rapidly. A balanced corpus of 9,986 phishing emails was assembled, comprising 4,986 emails generated by three modern LLMs (GPT-4.1, DeepSeek 3.2, and Llama 3.3 70B) across five thematic categories, and 5,000 human phishing emails sampled in a stratified manner from five public sources. Seventeen stylometric features were extracted, and Logistic Regression and XGBoost classifiers were evaluated under intra-model, cross-model, threshold-recalibrated, cross-dataset, and aggregated-pool settings. Intra-model F1 scores reached 0.96 under stratified cross-validation and 0.999 on held-out splits used for the cross-model matrix. However, cross-model F1 dropped by 28.0 percentage points under the default decision threshold of 0.5. Notably, the area under the receiver operating characteristic curve remained above 0.96 in every off-diagonal cell, indicating that discriminative information is preserved even though the decision threshold is generator-specific. Recalibrating the threshold on a small target subset reduced the gap to 4.0 percentage points (an 86% reduction), and an aggregated-pool detector achieved F1 = 0.997 on each generator. This work reframes cross-model phishing detection from a problem of model incompatibility to one of practical calibration, and provides two deployable solutions, threshold recalibration on a small target slice and aggregated-pool training, along with a publicly released multi-LLM corpus.

## 1 Introduction

Phishing remains the primary method for credential theft and initial access in enterprise environments. According to Anti-Phishing Working Group (2025), 853,244 phishing attacks were reported in the fourth quarter of 2025, following 1,130,393 cases in the second quarter and 892,494 in the third quarter, indicating that phishing activity has remained consistently high over time. Credential abuse constitutes 32% of breaches involving the human element. Phishing overlaps substantially with credential theft, as pretexting often leads to the subsequent misuse of stolen credentials (Verizon, 2025).

At the same time, large language models (LLMs) have substantially lowered the barrier to producing convincing phishing content. Unlike traditional phishing emails, which frequently contain spelling errors, awkward phrasing, or inconsistent formatting, AI-generated messages can closely replicate legitimate business correspondence with improved fluency and contextual awareness. Opara et al. (2025) demonstrated that AI-generated phishing created with GPT-4o can evade major spam filters. A stylometric approach using 47 features and XGBoost achieved 96% accuracy in distinguishing AI-generated phishing emails from legitimate emails. Related research on human-versus-LLM text classification indicates that lexical, grammatical, syntactic, and punctuation patterns are highly informative for detection (Opara, 2024).

These advancements pose substantial challenges for defenders. As phishing content becomes more sophisticated and model-generated, conventional filters that rely on surface-level anomalies may fail to detect an increasing proportion of attacks. Concurrently, research on AI-generated text detection shows that strong in-domain performance does not guarantee robust out-of-distribution results. Recent studies report that LLM-generated text detectors generalize poorly to new domains and that performance is highly dependent on model architecture, especially when evaluated across previously unseen domains or generators (Borile and Abrate, 2025).

This study examines the cross-model transferability of stylometric phishing detectors. The task is framed as authorship attribution within the phishing category: both training and evaluation samples consist of phishing emails, and the classifier distinguishes human-written phishing from LLM-generated phishing, rather than differentiating phishing from legitimate communication. Four research questions are formulated:

- RQ1. To what extent do stylometric detectors trained on phishing generated by one LLM generalize to phishing generated by other LLMs?
- RQ2. Is any observed gap attributable to insufficient class separation or to a mismatch in the decision threshold, and could an operator address this with a small labeled validation set?
- RQ3. Which stylometric features remain stable across LLMs, and which are specific to individual models?
- RQ4. Does pooling phishing emails from multiple LLMs into a single training set produce a robust universal detector?

To address these questions, a balanced corpus of phishing emails was generated by three modern LLMs across five thematic categories: banking, parcel delivery, IT support, tax/IRS, and human resources. This corpus is combined with five publicly available human phishing corpora. We compare two classifier families under five experimental conditions: Logistic Regression on z-scored stylometric features, and XGBoost on the same feature set.

The novelty of this work lies primarily in its methodological and empirical aspects rather than in its architecture. Established classifiers are employed to ensure the interpretability of the comparison. The main contribution lies in the experimental design's insights into cross-model behavior, which has not been previously investigated. Specifically, this paper makes five distinctive contributions, each addressing a gap in the existing literature on AI-generated phishing detection:

- A balanced multi-LLM phishing corpus. We release 4,986 phishing emails generated by three modern LLMs spanning distinct training paradigms (a commercial frontier model, an open-weight model, and an efficiency-optimized alternative) together with 5,000 stratified human phishing emails. To our knowledge, no prior public corpus has covered three contemporaneous LLMs at this scale.
- The first systematic cross-model evaluation of stylometric phishing detectors. We quantify, using a 3 × 3 transferability matrix, how much detection performance degrades when the phishing generator changes between training and evaluation, and we show that the default-threshold gap is large (28.0 percentage points in F1).
- A discriminative-versus-calibration decomposition of the transferability gap. We show that the area under the ROC curve stays above 0.96 in every off-diagonal cell, isolating the failure mode as a threshold-calibration phenomenon rather than a loss of discriminative information. To our knowledge, this decomposition has not been reported in the context of stylometric phishing detection.
- Identification of stable and model-specific stylometric features. A SHAP analysis identifies two features that remain in the top five across all three generators (politeness density and type-token ratio) and three that are specific to the open-weight Llama (verb ratio, first-person pronoun ratio, and sentence length), providing actionable guidance for detector design.
- An operationally deployable baseline. An aggregated-pool detector trained on the union of all three LLMs reaches F1 = 0.997 on each individual generator, offering a concrete recipe for security-operations-center deployment without per-LLM retraining.

The combined significance is twofold. For research, the threshold-calibration finding reframes the cross-model problem from one of fundamental model incompatibility to one of practical calibration, opening a new line of work on lightweight adaptation of stylometric detectors. For practice, the two deployable remedies (threshold recalibration on a small target slice and aggregated-pool training over multiple LLMs) reduce the cost of keeping a stylometric detector current as new LLMs emerge from full retraining on a few hundred labeled samples, which is feasible within hours of operational analyst work.

The remainder of the paper is organized as follows. Section 2 reviews related work. Section 3 presents the methodology. Section 4 reports the experimental results. Section 5 discusses implications and limitations. Section 6 concludes.

## 2 Related work

### 2.1 Phishing email detection: traditional approaches

Traditional phishing detection methods have primarily utilized three categories of techniques: rule-based and blacklist filters, URL- and header-based features, and machine learning over textual content (Salloum et al., 2022; Asiri et al., 2023; Bountakas et al., 2021). Existing research uses a diverse range of public corpora, including the Nazario phishing archive (Nazario, 2007), the CEAS-08 anti-spam challenge dataset (CEAS, 2008), the Enron email collection (Klimt and Yang, 2004), and the TREC public spam tracks (Cormack, 2007). Each dataset was collected under distinct conditions and labeling protocols. This heterogeneity is itself a methodological concern, because detectors trained on one corpus may not generalize to phishing styles present in others. These approaches share an additional weakness when confronted with LLM-generated content: they were tuned against phishing, exhibiting characteristic markers of non-native authorship, such as spelling mistakes, awkward phrasing, and template repetition, which modern LLMs largely eliminate. Several recent studies report substantial performance degradation when conventional detectors are evaluated against LLM-generated phishing (Heiding et al., 2024; Roy et al., 2024).

### 2.2 LLM-generated phishing and prior detection research

The dual use of LLMs in cybersecurity has prompted extensive research into both their offensive applications for generating phishing content and their defensive applications for detection. On the offensive front, Roy et al. (2024) demonstrated at IEEE S&P 2024 that ChatGPT, GPT-4, Claude, and Bard can be prompted to generate functional phishing websites and emails using both direct and evasive prompting strategies. Heiding et al. (2024) compared phishing emails crafted by GPT-4 with those produced by human red teamers in a controlled study with 112 participants, finding click-through rates of 30%–44%, comparable to or exceeding generic baselines. Heiding et al. (2026) extended this line with fully AI-automated spear-phishing campaigns, reporting 54% click-through rates and a 50 × cost reduction relative to human-equivalent campaigns.

On the defensive side, related research has focused on detecting AI-generated phishing using both LLM-based and stylometric approaches. Opara et al. (2025) introduced a 47-feature stylometric pipeline trained on 63 GPT-4o phishing emails and matched legitimate emails, achieving 96% accuracy and an AUC of 0.99 with XGBoost. This study represents the most direct methodological predecessor to the present work, and the intra-model results here extend those findings to three generators. Koide et al. (2026) developed ChatSpamDetector, a GPT-4-based detector that achieves 99.70% accuracy on a comprehensive phishing dataset, provides interpretable reasoning for its decisions. Chataut et al. (2024) evaluated the phishing detection capabilities of GPT-3.5, GPT-4, and a customized ChatGPT, reporting varying levels of success across the three models. In addition, Gore et al. (2023) discussed augmented-intelligence pipelines in which LLMs assist analysts rather than replace them, providing a defensive complement to the offensive-focused studies.

Collectively, these studies demonstrate that AI-generated phishing can be detected with high accuracy under intra-LLM conditions, where the same generator is used to produce the corpus and often to evaluate the detector. However, an unresolved question remains: whether such detectors generalize across different generators and what characterizes any resulting generalization gap. This paper addresses that gap through a systematic cross-model evaluation.

### 2.3 Stylometric detection of AI-generated text

Stylometric analysis, the quantitative study of writing style, has been extensively utilized in authorship attribution and forensic linguistics. Its application to the detection of AI-generated text began with early investigations into GPT-2 and GPT-3 outputs, where features such as type-token ratio, sentence-length distributions, and function-word frequencies distinguished machine-generated from human-written prose (Opara, 2024). In the context of phishing, Opara et al. (2025) introduced a comprehensive set of forty-seven stylometric features, categorized as lexical, syntactic, stylistic, and tonal. When applied to a dataset of sixty-three GPT-4o phishing emails and matched legitimate emails, their XGBoost classifier achieved 96% accuracy and an AUC of 0.99. Imperative-verb count, clause density, and first-person pronoun usage were identified as the most influential features. The dataset was made publicly available on Kaggle and has since become a reference point for subsequent research.

Recent complementary studies have examined AI-text detection beyond the phishing domain. Broader surveys of LLMs in cybersecurity indicate that detection robustness across different generators remains an unresolved challenge (Zhang et al., 2025; Xu et al., 2025). The systematic literature review by Zhang et al. (2025) highlights single-source training as a recurring limitation of LLM-based detectors, thereby motivating multi-generator evaluations such as those conducted in the present study.

### 2.4 Cross-model and generalization studies

The issue of whether AI text detectors generalize across different generative models has been addressed primarily in the broader literature on machine-generated text detection rather than in the specific context of phishing. Li et al. (2024) introduced MAGE, a benchmark encompassing ten domains and texts generated by 27 distinct LLMs, and demonstrated that the performance of mainstream detectors declines significantly under out-of-distribution conditions, especially when the test text originates from an unseen generator. Their analysis quantifies the reduction in the linguistic gap between human and AI-generated text as the generator varies, thereby complicating detection. These results support the hypothesis that single-source training leads to detectors with limited generalizability. However, MAGE focuses on neural detectors that operate directly on text, rather than on feature-engineered stylometric pipelines, and evaluates general open-domain content rather than phishing emails. To date, the transferability of stylometric detectors across LLMs has not been systematically investigated in the phishing context, nor has the performance gap been analyzed in terms of discriminative and calibration components. Addressing these gaps constitutes the primary contribution of the present paper.

## 3 Methodology

### 3.1 Experimental design overview

The study follows a five-stage pipeline: (i) collection and stratified sub-sampling of human phishing corpora; (ii) generation of LLM phishing emails using a controlled prompting protocol across three models and five thematic categories; (iii) extraction of seventeen stylometric features; (iv) training and evaluation of classifiers under five experimental conditions; (v) feature-importance analysis via SHAP. Each component is described in detail below.

### 3.2 Data sources

#### 3.2.1 Human phishing corpora

To construct the human side of the corpus, we combined six publicly available phishing and email datasets that have been widely used as benchmarks in the spam and phishing detection literature: CEAS-08 (CEAS, 2008), the Nazario phishing corpus (Nazario, 2007), the Nigerian Fraud dataset (Radev, 2008), TREC-07 (Cormack, 2007), the lingspam corpus (Sakkis et al., 2003), and a fraud-labeled subset of the Enron email collection (Klimt and Yang, 2004). Each dataset was loaded directly from its CSV source and normalized to a common schema (*subject, body, label*). Field names varied slightly across sources (for example, lingspam uses *message* for the body field, and the Enron release uses capitalized *Subject, Body*, and *Label*), and were mapped to the unified schema before any further processing.

After normalization, the combined pool contained 56,728 phishing-positive emails (label = 1). Four cleaning and sub-sampling steps were applied. First, we excluded the lingspam corpus due to its outlier-length distribution and limited phishing volume compared to our other sources. Second, we filtered by token length to the range 30 ≤ tokens ≤ 500, aligning the human side with realistic short-form email content and with the length distribution of the LLM-generated emails described in Section 3.3.2. Third, we applied stratified sub-sampling with explicit quotas per source: 1,500 from CEAS-08, 1,500 from TREC-07, 1,000 from Nazario, 750 from Nigerian Fraud, and 250 from Enron. Fourth, exact-duplicate emails were removed across sources. The final human corpus contains 5,000 phishing emails with a mean length of 164.8 tokens (median 115, std 127.1). The stratification quotas were chosen to balance representation across sources while keeping the dominant contributors (CEAS-08 and TREC-07) from overwhelming the smaller corpora.

#### 3.2.2 LLM-generated phishing

Three contemporary LLMs are employed to generate phishing emails:

- GPT-4.1 (OpenAI), representing the OpenAI commercial frontier at the time of generation.
- DeepSeek 3.2 (DeepSeek), representing an alternative training paradigm with documented efficiency-oriented design and a distinct geographic ecosystem.
- Llama 3.3 70B (Meta), representing the open-weight community as a generative resource.

All three models are accessed via a unified OpenAI-compatible endpoint (Azure AI Foundry), which standardizes request semantics and parameter handling across models. The selection of models from distinct training lineages is intentional, reflecting the diversity likely to be encountered in adversarial scenarios.

### 3.3 Phishing email generation protocol

The generation was conducted entirely within a declared defensive security-research framing, in line with standard practice in the LLM-phishing literature (Roy et al., 2024; Heiding et al., 2024, 2026). The system prompt explicitly identified the task as producing synthetic phishing examples for an academic study on AI-generated social engineering, stated that the outputs would be released as a labeled corpus for training detection models, and noted that the messages would never be sent to real recipients. No jailbreak, malicious-agent roleplay, prompt injection, obfuscation, or guardrail-circumvention strategy was used. The complete system prompt and the per-category user template are reproduced in Supplementary material.

#### 3.3.1 Thematic categories

Five thematic categories are used: banking (account verification, security alerts), parcel delivery (failed delivery, customs fees), IT support (password reset, mailbox quota), tax/IRS (refund claims, audit notices), and human resources (compensation memos, benefits enrolment). These categories cover the most prevalent phishing themes in industry reports.

#### 3.3.2 Prompting strategy

A single prompt template is used across all three models to enable cross-model comparability. It consists of a fixed system prompt and a parameterized user prompt populated with three slots sampled from per-category lists. Both are reproduced below verbatim.

##### 3.3.2.1 System prompt

You are a senior security researcher producing realistic phishing email examples for an academic study on AI-generated social engineering. All outputs are used solely for defensive research, will be released as a labeled corpus for training detection models, and will never be sent to real recipients. Generate convincing examples that reflect what a skilled attacker might compose. Do not include any disclaimers, warnings, or meta-commentary about the email being a sample or a test.

##### 3.3.2.2 User prompt template


Generate one phishing email for the academic corpus.
    Category: {category}
Sender persona: {sender_persona}
Pretext/hook: {hook}
Desired target action: {target_action}
    Constraints:

- Length between 80 and 350 words.
- Realistic but generic; do not use real company names, real employee names, real domains, or real telephone numbers. Use plausible placeholders such as “YourBank,” “support@your-bank-portal.com,” “+1-800-160-0947.”
- Include a Subject line on the first output line, prefixed exactly with “Subject: .” After the subject, leave a blank line, then write the email body.
- Do NOT include any preamble, explanation, disclaimer, or markdown formatting. Output only the email itself, starting with “Subject:.”

##### 3.3.2.3 Per-category slot lists

For each category, four options are provided for each slot (sender persona, hook, and target action), yielding up to 4 × 4 × 4 = 64 unique combinations per category and 320 per model. Table 1 lists the complete contents of every slot.

**Table 1:** Per-category slot lists used in the user prompt template.

Category	Sender persona	Hook	Target action
Banking	A major retail bank's security team; an online banking fraud-prevention department; a credit-card issuer's risk-control unit; a wealth management advisor	An unusual login attempt from a foreign country; a temporary account suspension pending verification; a large pending transfer that needs confirmation; an expiring security certificate on the account	Click a link to verify the account; reply with the last four digits of the card; open an attached secure document; call a fraud hotline number
Parcel delivery	A major international courier service; a postal service customs department; a same-day delivery dispatcher; a returns-processing center	A parcel held due to an unpaid customs fee; a failed delivery attempt requiring rescheduling; an address mismatch on a high-value shipment; a missing signature on a recorded-delivery item	Click a link to schedule redelivery; pay a small handling fee online; confirm the delivery address; download a delivery slip attachment
IT support	The corporate IT helpdesk; the company's identity and access management team; the Microsoft 365 administrator; the cloud-storage service provider	An expiring password that must be reset within 24 h; a mailbox approaching its storage quota; a security incident requiring immediate password rotation; a pending multi-factor authentication enrollment	Click a portal link to update credentials; verify identity through an external form; install a mandatory security update; review and approve a sign-in request
Tax/IRS	The Internal Revenue Service tax-refund unit; a national tax authority compliance office; a tax-filing assistance service; a refund-processing center	An unclaimed tax refund pending direct deposit; a discrepancy in the most recent tax filing; a final notice before a wage garnishment; additional documentation required to release a payment	Log in through a portal to claim the refund; fill in a secure form with bank details; open an attached PDF with the discrepancy details; call a dedicated case-handler line
HR	The company's Human Resources department; the payroll administrator; the benefits-enrollment coordinator; the internal compliance and ethics office	A revised compensation document requiring acknowledgment; a benefits-enrollment deadline that closes today; an updated employee handbook that must be signed; a confidential staffing-reorganization memo	Click a link to view the document on the HR portal; log in to confirm bank details for the next payroll; open and sign an attached PDF; complete a short verification form

Each of the three slots is sampled independently using a fixed random seed. The generation parameters remain constant across all models: temperature is set to 0.7, top-*p* to 0.95, and the maximum number of tokens to 500.

#### 3.3.3 Quality control and filtering

The 4,995 raw outputs (1,665 per model) were passed through a five-stage automated pipeline implemented in a Python script on the Supplemental material. The pipeline applies the following filters in sequence, recording the number of removals at each stage:

1. **Empty body**. Outputs whose body field is empty after trimming whitespace are removed. Result: 0 removed.
2. **Empty subject**. Outputs that do not produce a The subject line on the first non-empty line, indicating that the model failed to follow the requested format, is removed. Result: 0 removed.
3. **Refusal detection**. Outputs matching any of an explicit set of case-insensitive refusal patterns are removed. Result: 0 removed.
4. **Length filter**. Outputs outside the range 30 ≤ tokens ≤ 500 are removed, matching the bound applied to the human corpus. Result: 0 removed.
5. **Near-duplicate removal**. Outputs are normalized (lowercased, whitespace-collapsed), and the first 500 characters are used as a deduplication key within each (model, category) bucket. Result: 9 removed, all originating from Llama 3.3 70B and distributed across the categories IT support (4), HR (3), banking (1), and tax/IRS (1).

The merge pipeline excludes human curation; filtering is fully automated, deterministic, and reproducible from raw CSV outputs using the released script. Two forms of human inspection were applied to the corpus. During development, representative outputs across all categories and models were inspected to confirm that the generated emails were plausibly phishing-like and that no systematic failure mode (such as refusals, format breakage, or off-topic content) was missed by the automated filters. Additionally, a stratified random sample of 60 emails (4 per model-category combination) was reviewed to verify that prompt-level category labels matched the manifest content. No further removal was performed beyond the automated pipeline.

After filtering, the final LLM corpus comprises 4,986 emails. (GPT-4.1: 1,665; DeepSeek 3.2: 1,665; Llama 3.3 70B: 1,656). The token-length distribution is well-centered (mean 193.3, median 193, std 24.9, min 117, max 294), comparable in scale to the human corpus, and falls within the range of realistic email lengths.

#### 3.3.4 Representative examples by category

To facilitate direct inspection of the generated corpus structure, Table 2 summarizes the metadata of one representative LLM-generated phishing email per category, drawn from the three generators used in this study, and the verbatim text of each example follows. URLs, email addresses, and phone numbers are synthetic placeholders generated by the prompt; no real domains, addresses, or phone numbers appear in the corpus.

**Table 2:** Metadata of the five representative LLM-generated phishing examples reproduced below.

Category	Model	Sender persona	Hook	Target action
Banking	GPT-4.1	A wealth management advisor	An expiring security certificate on the account	Click a link to verify the account
Parcel delivery	GPT-4.1	A same-day delivery dispatcher	A failed delivery attempt requiring rescheduling	Pay a small handling fee online
IT support	Llama 3.3 70B	The cloud-storage service provider	A mailbox approaching its storage quota	Review and approve a sign-in request
Tax/IRS	DeepSeek 3.2	The Internal Revenue Service tax-refund unit	An unclaimed tax refund pending direct deposit	Log in through a portal to claim the refund
HR	DeepSeek 3.2	The benefits-enrollment coordinator	A confidential staffing-reorganization memo	Log in to confirm bank details for the next payroll

##### 3.3.4.1 Example 1: banking (GPT-4.1)

Subject: Immediate Action Required: Account Security Certificate Expiring Soon

Dear Valued Client,

I am reaching out as your designated wealth management advisor to inform you of a recent update concerning your YourBank online account. Our security monitoring systems have detected that the digital security certificate associated with your account is set to expire within the next 48 h.

To ensure uninterrupted access and to safeguard your financial information, it is essential to verify your account and renew your security certificate as soon as possible. Failure to do so may result in temporary restrictions on your online banking services, including access to portfolio management, fund transfers, and account statements.

Please take a moment to complete the verification process by following the secure link below:

https://your-bank-portal.com/secure/verify-account

For your protection, do not share your login details with anyone. If you do not complete the verification within the indicated time frame, your account may be automatically flagged for additional review.

If you have any questions or require assistance, please contact our support team at support@your-bank-portal.com or call us at +1-800-386-1846.

Thank you for your prompt attention to this important matter.

Sincerely,

Jordan Matthews Wealth Management Advisor YourBank

##### 3.3.4.2 Example 2: parcel delivery (GPT-4.1)

Subject: Action Required: Reschedule Your Same-Day Parcel Delivery

Dear Customer,

We attempted to deliver your package today as requested, but unfortunately, no one was available to receive it at your address. To ensure the safe and timely delivery of your parcel, we require you to reschedule the delivery at your earliest convenience.

Due to storage and reprocessing costs, a small handling fee of $2.75 is required to arrange a new delivery slot. Please complete the rescheduling process by clicking the link below and submitting the fee through our secure portal:

https://deliveryportal-support.com/reschedule?id=83927

Your package will remain on hold for the next 48 h. If we do not receive your rescheduling request within this period, your parcel may be returned to the sender.

If you have any questions or need assistance, please reply to this email or contact our support team at support@deliveryportal-support.com.

Thank you for choosing our same-day delivery service.

Best regards, Dispatch Services Team Same-Day Delivery Support +1-800-470-6795

##### 3.3.4.3 Example 3: IT support (Llama 3.3 70B)

Subject: Action Required: Mailbox Storage Quota Approaching

Dear valued customer,

We are writing to inform you that your mailbox is approaching its storage quota. As your cloud storage service provider, we want to ensure that you have uninterrupted access to your emails and files. Our system has detected that your current mailbox size is 99% of the allocated limit, and we recommend taking immediate action to avoid any potential disruptions.

To assist you in managing your mailbox storage, we have initiated a sign-in request to verify your account activity. Please review and approve this request to ensure the security and integrity of your account. This verification process will also help us to better understand your storage needs and provide you with personalized recommendations for optimizing your mailbox.

To approve the sign-in request, simply click on the following link: https://cloud-storage-portal.com/signin-verification. You will be prompted to enter your login credentials and confirm the request. If you have any questions or concerns, please do not hesitate to contact our support team at support@cloud-storage-portal.com or call us at +1-800-456-1753.

We appreciate your prompt attention to this matter, and we are committed to providing you with the best possible service. Thank you for choosing our cloud storage services.

Best regards, The Cloud Storage Team

##### 3.3.4.4 Example 4: tax/IRS (DeepSeek 3.2)

Subject: Final Notice: Unclaimed Tax Refund - Action Required

Dear Taxpayer,

Our records indicate you have an unclaimed tax refund from the previous filing year. The Internal Revenue Service has attempted to contact you regarding this pending direct deposit, but we have not received the necessary verification to release your funds.

Your refund amount is listed as $847.62 and is being held securely. To claim your refund and initiate the direct deposit, you must verify your identity and banking information through our secure online portal. This is a standard procedure to prevent fraud and ensure funds are delivered to the correct recipient.

Please use the button below to access the secure verification portal. You will be asked to confirm your Social Security Number and current banking details for deposit.

[ VERIFY AND CLAIM REFUND ]

For security, this link will expire in 72 h. If you do not complete verification, your refund will be reallocated to the U.S. Treasury, and you may need to file additional paperwork to claim it in the future.

If you have questions, you may contact our Refund Support line at +1-800-555-4235. Please have your Taxpayer ID ready.

Sincerely, Internal Revenue Service Tax Refund Processing Unit

##### 3.3.4.5 Example 5: HR (DeepSeek 3.2)

Subject: Confidential: Staffing reorganization & Urgent Payroll Verification

Dear Team Member,

This email is being sent to all employees as part of a confidential, company-wide staffing reorganization initiative. As announced in our recent all-hands meeting, these strategic adjustments are designed to enhance operational efficiency for the upcoming fiscal year.

To ensure seamless continuity of all payroll and benefits functions during this transition, we are conducting a mandatory verification of employee banking details. This is a critical step to prevent any potential disruption to your salary deposits.

You are required to confirm your information via the secure HR portal within the next 48 hours. Please click the link below to log in and review your current bank details on file. You will be asked to re-enter your credentials for security verification.

[Secure HR Portal Login]

If your details are correct, simply select “Confirm.” If updates are needed, you will be guided through the correction process. For security, this link is unique to this verification campaign and will expire after the deadline.

For immediate assistance, please contact the HR Support Desk at +1-800-257-4367.

Thank you for your prompt attention to this important matter.

Sincerely,

Patricia Reynolds Benefits & Enrollment Coordination Human Resources Department

### 3.4 Stylometric feature extraction

Seventeen features are extracted from each email, selected from the broader feature set of Opara et al. (2025) and organized into four linguistic families: lexical (4), syntactic (4), stylistic (5), and phishing-specific (4). The full list is given in Table 3.

**Table 3:** Stylometric feature subset (*n* = 17) extracted from each email.

Category	Feature	Linguistic dimension
Lexical	Type-token ratio (TTR)	Lexical diversity
Lexical	Mean word length (chars)	Surface lexical signal
Lexical	Mean sentence length (tokens)	Verbosity/pacing
Lexical	Yule's *K*	Vocabulary concentration
Syntactic	Clause density	Syntactic complexity
Syntactic	Noun ratio	Nominal style
Syntactic	Verb ratio	Action orientation
Syntactic	Mean dependency-parse depth	Structural complexity
Stylistic	Imperative count	Urgency/call-to-action
Stylistic	First-person pronoun ratio	Authorial voice
Stylistic	Second-person pronoun ratio	Reader address
Stylistic	Politeness density	Tonal register
Stylistic	Urgency density	Pressure tactic
Phishing	URL density	Link payload
Phishing	Call-to-action density	Conversion pressure
Phishing	Authority appeal density	Social-engineering lever
Phishing	Time-pressure density	Deadline tactic

This subset was selected a priori, before any classifier training, based on three criteria: (i) features must be computable from the email body alone, without reliance on external network resources. This criterion excludes URL-reputation, DNS, and header features, which conflate content authorship with delivery infrastructure; (ii) language-model-agnostic, so that the same extraction code applies across all three LLMs without per-model tuning; and (iii) representative of the four linguistic families most relevant to phishing persuasion. No data-driven feature selection was performed: the SHAP analysis reported in Section 4.7 is a post hoc interpretation, not feature selection. This design choice avoids the optimistic performance bias that arises when features are filtered using the same data later used for evaluation.

Lexical features (4) capture vocabulary richness and surface lexical patterns. The type-token ratio (TTR) is the number of unique word types divided by the total number of word tokens and serves as a standard measure of lexical diversity. Lower TTR values typically indicate repeated vocabulary. Mean word length in characters is a surface indicator of register, as formal text tends to contain longer words. Mean sentence length in tokens measures verbosity and pacing. Yule's K is a vocabulary-concentration statistic that, unlike TTR, is theoretically independent of text length and quantifies the frequency of word reuse.

Syntactic features (4) characterize sentence structure. Clause density is defined as the average number of clauses per sentence, calculated by dividing the count of ROOT, ccomp, advcl, relcl, and xcomp dependency relations by the number of sentences. Noun ratio and verb ratio represent the fractions of part-of-speech tags assigned to each class, respectively. These ratios are computed using spaCy's POS tagger. Mean dependency-parse depth is the average distance from each token to the ROOT of its sentence in the dependency tree; greater depth indicates more nested syntactic structure. syntactic structure.

Stylistic features (5) target tone and register. Imperative count refers to the number of sentences whose root verb appears in base form, which is a common indicator of urgency or a call to action. First-person and second-person pronoun ratios are calculated as the count of pronouns in each class divided by the total token count, reflecting authorial voice and reader address, respectively. Politeness density and urgency density are determined by counting matches against compact lexicons of polite markers (e.g., please, kindly, thanks, and regards) and urgency markers (e.g., urgent, immediately, asap, and deadline), normalized per 100 words. The full lexicons are released in the Supplementary material.

Phishing-specific features (4) capture persuasion mechanisms identified in the phishing literature. URL density measures the number of URL-like patterns per 100 words. Call-to-action density quantifies action-eliciting verbs and phrases (e.g., click, verify, and confirm). Authority-appeal density counts references to authority figures and institutions used to legitimize the message (e.g., IRS, police, administrator, and official). Time-pressure density measures temporal-deadline expressions (e.g., 24 h, before, and expired). All four features are normalized per 100 words. The corresponding lexicons are provided in the Supplementary material.

Feature extraction is implemented in Python 3.11 using spaCy with the en_core_web_sm model for tokenization, POS tagging, and dependency parsing. The implementation and associated dictionaries are available in the project repository.

### 3.5 Classifiers

Two classifiers are evaluated. Logistic Regression with z-scored features serves as an interpretable linear baseline, with class_weight = 'balanced' to address the slight human/LLM imbalance. XGBoost (200 estimators, max depth 6, and learning rate 0.1) is used as the non-linear reference, replicating the configuration of Opara et al. (2025). Hyperparameters are not tuned beyond these defaults to ensure reproducibility. The selection of two classifiers from distinct families, specifically a linear model and a tree ensemble, enables the separation of effects attributable to modeling assumptions from those inherent to the feature space.

### 3.6 Evaluation tasks

All five tasks are framed as binary authorship attribution within phishing, with LLM-generated emails as the positive class and human-written emails as the negative class. No legitimate email is included; reported metrics should be interpreted accordingly.

- **Task A: Intra-model evaluation**. For each LLM, the human-written emails are combined with the corresponding LLM-generated emails to train a binary classifier that distinguishes human from synthetic content. Performance is evaluated using stratified five-fold cross-validation, providing the within-model baseline for RQ1.
- **Task B: Cross-model evaluation (default threshold)**. A 3 × 3 transferability matrix is constructed. For each ordered pair (*A, B*), a detector is trained on human emails together with emails generated by LLM-A, and evaluated on a test set composed of held-out human emails (20%) and emails generated by LLM-B. The default decision threshold of 0.5 is retained. Performance degradation in off-diagonal evaluations quantifies the transferability gap associated with cross-model generalization (RQ1).
- **Task B′: Cross-model evaluation with threshold recalibration**. This setting follows the same protocol as Task B, except that 30% of the evaluation LLM data, together with a matched subset of held-out human emails, is reserved as a calibration set for selecting the threshold that maximizes the F1-score. The remaining 70% is used exclusively for testing. This experiment separates the transferability gap into discrimination-related and calibration-related effects (RQ2).
- **Task C: Cross-dataset human verification**. To determine whether cross-model degradation may instead arise from shifts in the human-written data, a detector is trained using the human subset from dataset A together with emails generated by all LLMs, and then evaluated on the human subset from dataset B together with the same pooled LLM data. This procedure is repeated for all pairs of human datasets.
- **Task D: Aggregated-pool detector**. A detector is trained on the combined outputs of all three LLMs against the human-written emails and subsequently evaluated on each individual LLM separately, thereby assessing the robustness of a generalized detector across generators (RQ4).

### 3.7 Metrics

To formalize the transferability gap discussed throughout the paper, let *M* be the 3 × 3 matrix of F1-scores obtained under the cross-model protocol of Task B, indexed by training generator *i* and evaluation generator *j*. The transferability gap is defined as the difference between the mean diagonal F1 and the mean off-diagonal F1:


$$\begin{array}{l} \Delta_{\text{F1}}=\frac{1}{3}\overset{3}{\underset{i=1}{\sum }}M_{ii}-\frac{1}{6}\overset{3}{\underset{\begin{array}{c} i,j=1\\ i\neq j \end{array}}{\sum }}M_{ij} \end{array}$$ (1)

The same definition applies to AUC-ROC and to the recalibrated matrix in Task B′. Under the default decision threshold of 0.5, Δ_F1_ = 0.28 (28.0 percentage points). Under threshold recalibration on a 30% target slice, Δ_F1_ = 0.040 (4.0 percentage points), an 86% reduction.

### 3.8 Reproducibility

All code, prompt templates, feature-extraction scripts, and evaluation pipelines are archived on Zenodo at https://doi.org/10.5281/zenodo.20250116. Random seeds are fixed at 42 for sampling, splitting, and model initialization. Exact replication of LLM outputs cannot be guaranteed because API providers may update model versions; we document model identifiers and API parameters at the time of generation.

## 4 Results

### 4.1 Dataset characterization

The combined corpus contains 9,986 emails: 5,000 human and 4,986 LLM (1,665 GPT-4.1, 1,665 DeepSeek 3.2, 1,656 Llama 3.3 70B). Table 4 reports descriptive statistics on the seventeen stylometric features selected a priori in Section 3.4, comparing human and LLM means.

**Table 4:** Mean values of selected stylometric features by class.

Feature	Human	LLM	Ratio (LLM/human)
TTR (lexical diversity)	0.677	0.610	0.90
Mean word length (chars)	4.764	5.255	1.10
Clause density	1.776	1.971	1.11
Imperative count	0.658	0.178	0.27
Politeness density	0.987	2.955	2.99
Urgency density	0.816	1.208	1.48
URL density	1.326	0.316	0.24
Call-to-action density	1.448	4.017	2.77
Authority density	0.234	0.707	3.02
Time-pressure density	0.464	1.304	2.81

Densities are reported per 100 words. The Ratio column shows the LLM/Human ratio; values above 1 indicate higher values for the LLM class, and values below 1 indicate higher values for the Human class.

**Figure 1:**
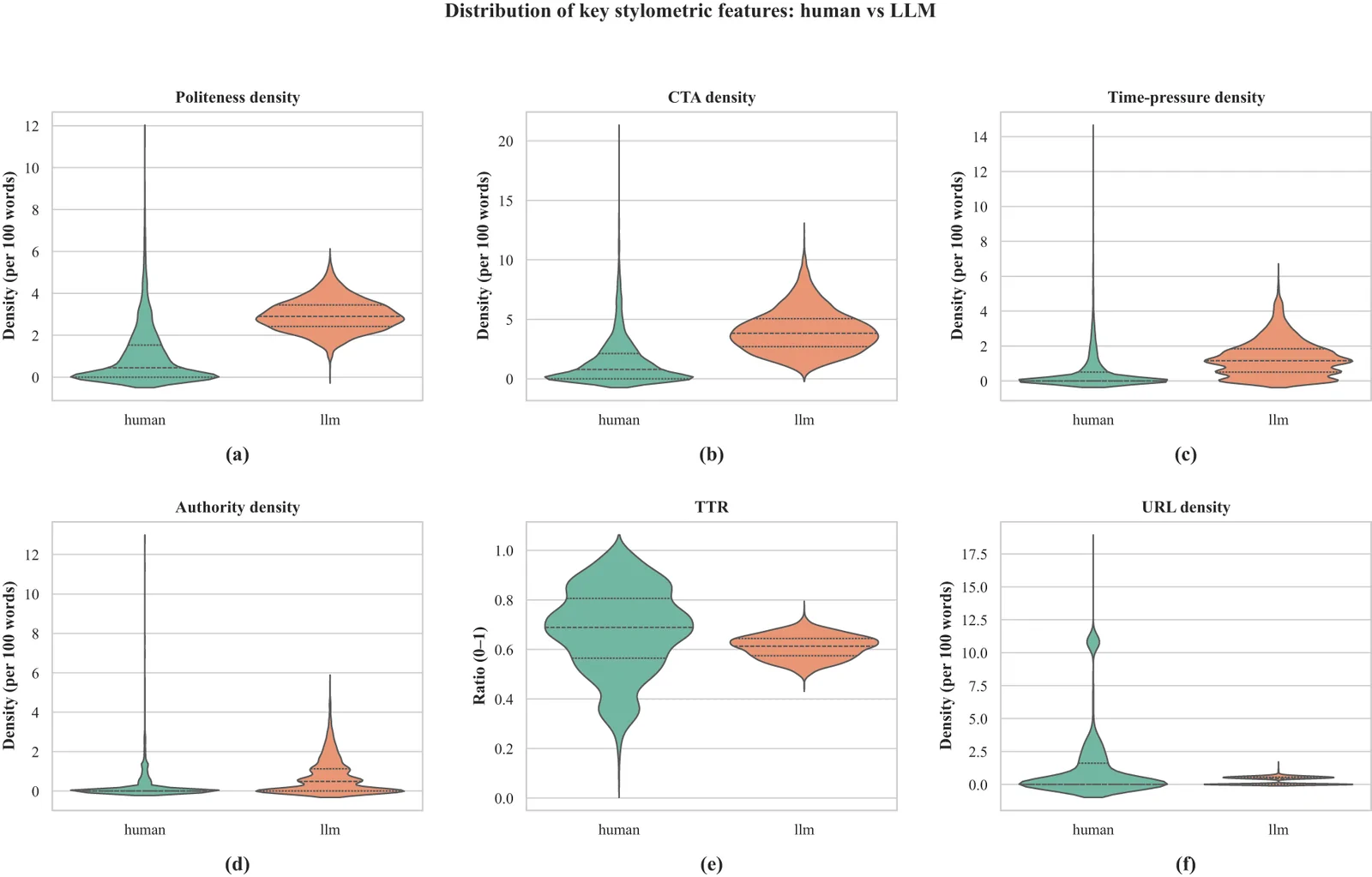
Distribution of six key stylometric features by class (human vs. LLM): **(a)** Politeness density; **(b)** CTA density; **(c)** Time-pressure density; **(d)** Authority density; **(e)** Ration (0–1); **(f)** URL density.

The two classes differ across several stylistic dimensions, with differences of sufficient magnitude to suggest strong classifier performance. Three groups of features demonstrate the most pronounced separation. Social-engineering markers—politeness density (LLM/Human ratio ≈2.99), call-to-action density (≈2.77), authority-appeal density (≈3.02), and time-pressure density (≈2.81)—are roughly three times more frequent in LLM phishing than in the human corpus. Lexical and syntactic markers show smaller but consistent shifts: LLM phishing exhibits lower lexical diversity (TTR ratio ≈0.90), longer words (ratio ≈1.10), and slightly denser clausal structure (ratio ≈1.11), a pattern consistent with prior reports of reduced lexical richness and increased syntactic uniformity in AI-generated text (Opara, 2024). By contrast, two features run in the opposite direction: imperative count (ratio ≈0.27) and URL density (ratio ≈0.24) are substantially higher in human phishing. The lower URL density in the LLM corpus reflects an explicit constraint placed on the generation prompts, which required plausible placeholder URLs rather than real domains; it should therefore be interpreted as a prompting artifact rather than as a stable stylistic signature of LLM output. All pairwise differences in Table 4 are statistically significant at *p* < 0.001 under a two-sided Mann–Whitney *U* test, as expected given the corpus size (*n*_human_ = 5, 000, *n*_LLM_ = 4, 986). Figure 1 shows the distributional patterns underlying these means using violin plots, highlighting the substantial separation between classes for the most discriminative features.

### 4.2 Task A: intra-model performance

**Figure 2:**
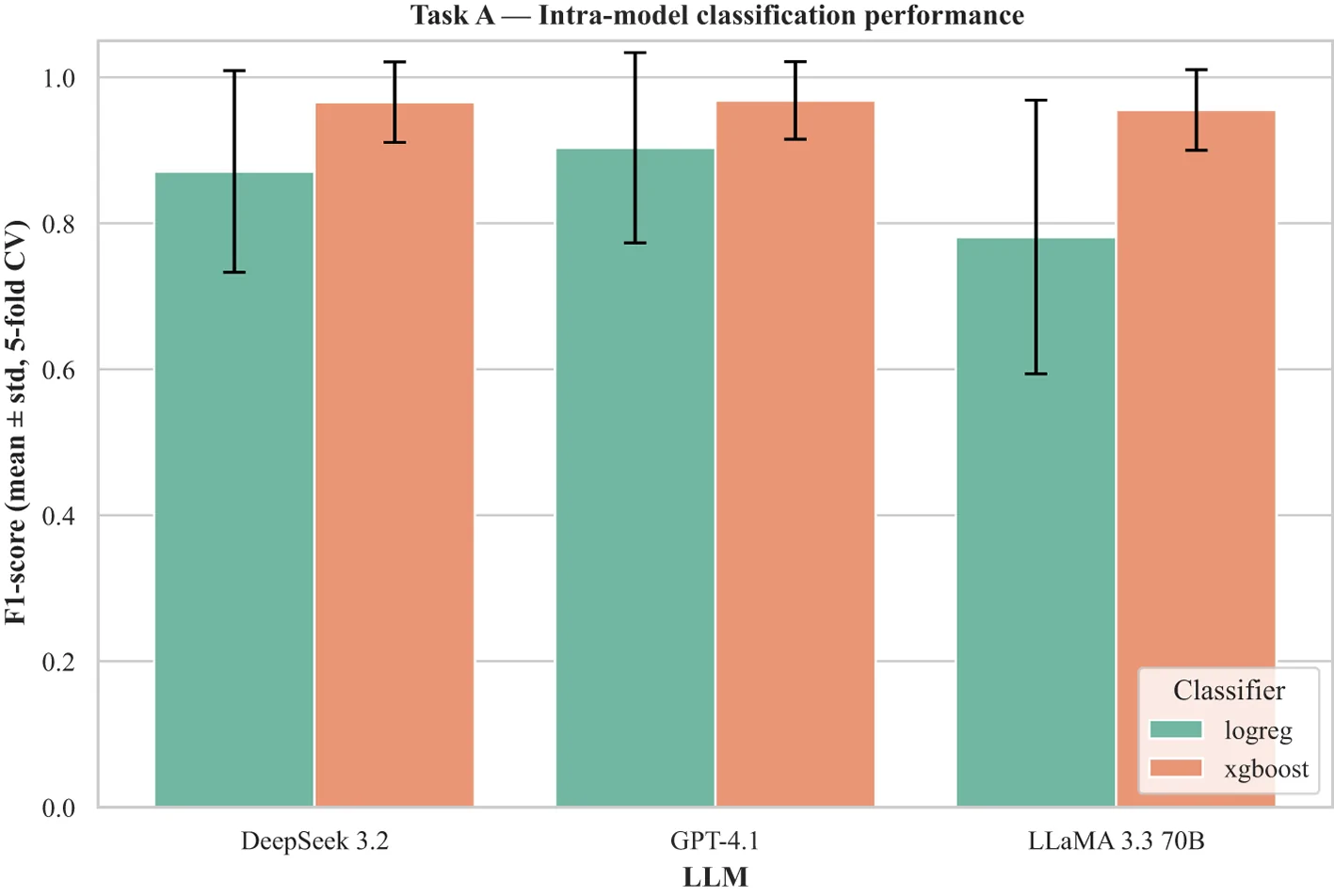
Intra-model F1 by classifier and LLM (mean ± std, 5-fold stratified CV).

Table 5 reports intra-model XGBoost classification metrics under stratified five-fold cross-validation. XGBoost on stylometric features achieves an F1 of 0.965 on GPT-4.1, 0.962 on DeepSeek 3.2, and 0.952 on Llama 3.3 70B, with AUC-ROC above 0.999 in all cases. Logistic Regression, shown in Figure 2, performs noticeably worse on Llama than on the other two models. The XGBoost-LogReg gap is largest for Llama, suggesting a more complex non-linear decision boundary in that model.

**Table 5:** Intra-model XGBoost classification metrics under stratified five-fold cross-validation.

Model	F1	AUC
GPT-4.1	0.9650	1.000
DeepSeek 3.2	0.9624	0.999
Llama 3.3 70B	0.9516	0.999

Predictions concatenated across folds.

These intra-model results replicate the high detection accuracy reported by Opara et al. (2025) for a single LLM, now demonstrated across three modern LLMs. This confirms that stylometric detection is a viable approach in fixed-model settings. Extending the analysis to three distinct generators, including the open-weight Llama 3.3 70B, constitutes a methodological contribution by broadening the validity of the original findings beyond a single commercial frontier model.

### 4.3 Error analysis

To enhance the F1-score with operational interpretability suitable for security-classification contexts, Table 6 presents per-LLM confusion matrices, sensitivity, and specificity for Task A, as well as pooled-detector metrics for Task D. Sensitivity (recall of the LLM class) remains consistently high, ranging from 99.15% for Llama 3.3 70B to 99.34% for GPT-4.1, demonstrating that the classifier rarely fails to identify LLM-generated emails. Specificity (recall of the human class) is also high, though slightly lower, spanning 96.94% (Llama) to 97.82% (GPT-4.1). The absolute number of misclassified emails per model is low: 109–153 false positives and 11–14 false negatives, out of approximately 6,665 evaluations. The pooled detector achieves a sensitivity of 1.000 and a specificity of 0.990 on each held-out LLM, with zero false negatives. in all three cases.

**Table 6:** Per-LLM confusion matrices and derived metrics under Task A (5-fold stratified cross-validation, predictions concatenated across folds).

Model	TN	FP	FN	TP	Sensitivity	Specificity	Precision	F1
GPT-4.1	4,891	109	11	1654	0.9934	0.9782	0.9382	0.9650
DeepSeek 3.2	4,883	117	12	1653	0.9928	0.9766	0.9339	0.9624
Llama 3.3 70B	4,847	153	14	1642	0.9915	0.9694	0.9148	0.9516
Aggregated-pool detector (Task D), evaluated on each held-out LLM								
Pool GPT-4.1	990	10	0	1665	1.0000	0.9900	0.9940	0.9970
Pool DeepSeek 3.2	990	10	0	1665	1.0000	0.9900	0.9940	0.9970
Pool Llama 3.3 70B	990	10	0	1656	1.0000	0.9900	0.9940	0.9970

TN, human correctly classified; FP, human classified as LLM; FN, LLM classified as human; TP, LLM correctly classified.

A qualitative analysis of the misclassified emails reveals consistent patterns. False positives, defined as human-written phishing classified as LLM-generated, are predominantly highly polished, templated phishing emails. These examples, drawn from the Nazario corpus, include USAA-style banking alerts, IT system upgrade notifications, and email quarantine warnings. Such emails display formal corporate vocabulary, comprehensive politeness markers, and structured calls to action, all of which are characteristic of LLM-generated phishing. Consequently, the misclassification reflects a genuine stylistic convergence between professionally crafted human phishing and LLM output, rather than a deficiency in the classifier.

False negatives, defined as LLM-generated phishing classified as human, exhibit a complementary pattern. These instances are concentrated in three thematic clusters: HR staffing reorganization memos, tax or IRS refund notifications, and customs-fee parcel notices. These clusters share a deliberately bureaucratic register, characterized by a neutral tone, formal sentence structure, absence of explicit urgency or call-to-action markers, and language closely modeled on authentic corporate or institutional communication. When LLMs are prompted to generate phishing in this register, the resulting text exhibits a stylometric signature more similar to human-written emails than to typical LLM-generated phishing. This observation has direct operational implications: adversaries can shift LLM-generated phishing across the stylometric decision boundary by specifying a bureaucratic style, indicating that defensive systems should be explicitly evaluated against this adversarial variant.

### 4.4 Task B and Task B′: cross-model transferability and threshold recalibration

**Figure 3:**
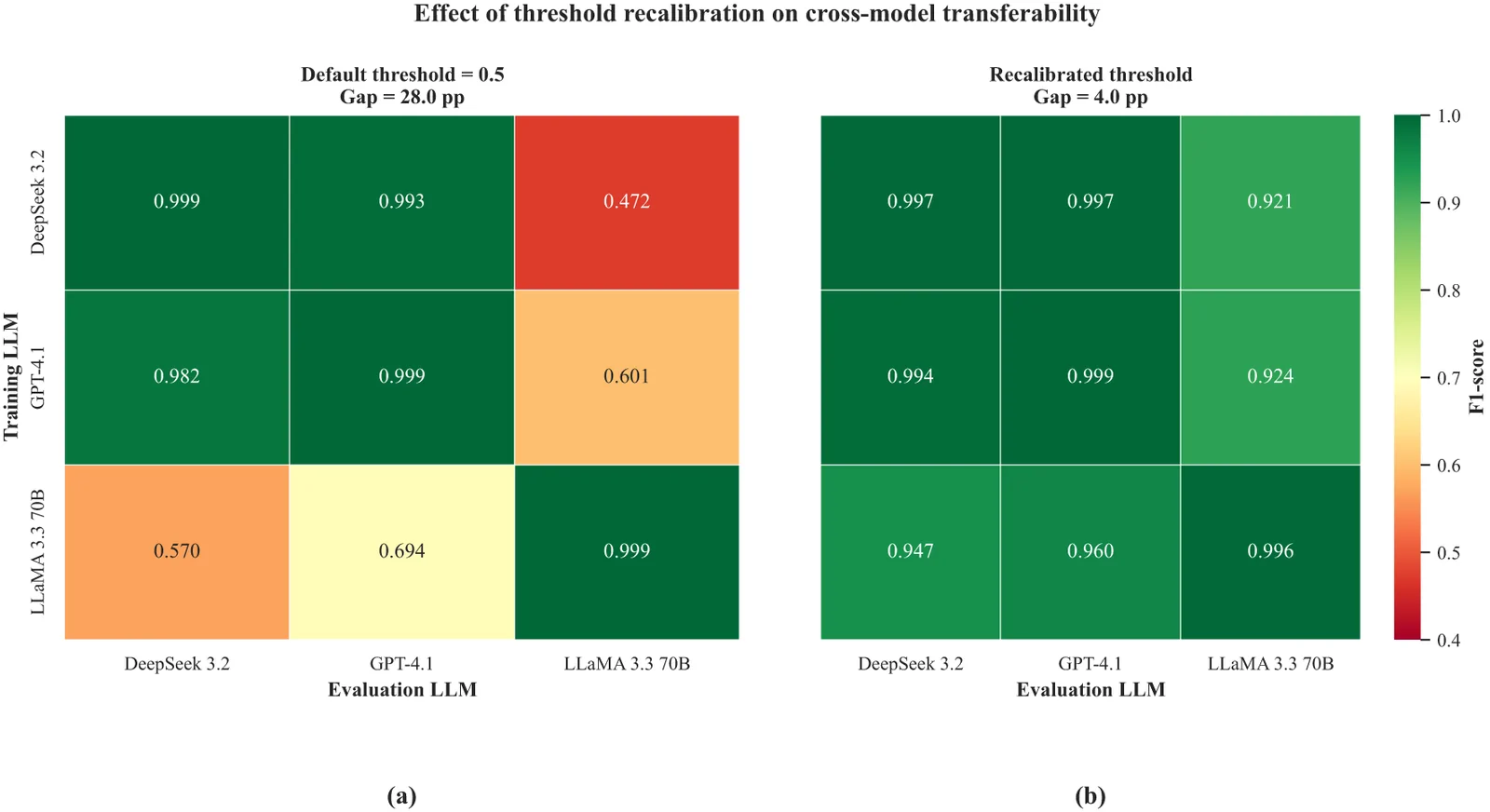
Cross-model transferability of stylometric detectors (F1-score). **(a)** Default threshold (0.5); transferability gap = 28.0 pp. **(b)** After threshold recalibration on 30% of the target LLM; gap = 4.0 pp.

Figure 3 presents the central result of the paper. The left panel shows the cross-model F1 matrix obtained with the XGBoost stylometric classifier and the default decision threshold of 0.5. The diagonal entries are above 0.999, but several off-diagonal cells fall sharply: detectors trained on DeepSeek 3.2 and evaluated on Llama 3.3 70B drop to F1 = 0.472, while training on GPT-4.1 and evaluating on Llama yields F1 = 0.601. Detectors trained on Llama also fail to generalize to the other two models, with F1 of 0.570 (DeepSeek) and 0.694 (GPT-4.1). The mean diagonal F1 is 0.999, the mean off-diagonal F1 is 0.719, and the transferability gap is 28.0 percentage points.

Critically, AUC-ROC remains above 0.96 in every off-diagonal cell of Figure 3a. This indicates that the discriminative information is preserved across models, but the decision threshold of 0.5 is no longer well-calibrated for the eval distribution. To test this interpretation, Task B′ replaces the default threshold with one tuned to maximize F1 on a 30% calibration slice of the target LLM. The right panel of Figure 3b shows the result: cross-model F1 increases substantially in every off-diagonal cell, with the weakest cell rising from 0.472 to 0.921. The mean off-diagonal F1 becomes 0.957, the diagonal mean is 0.997, and the transferability gap collapses from 28.0 to 4.0 percentage points, an 86% reduction.

This decomposition clarifies the practical implications for security operations: most observed transferability failures arise from calibration issues rather than discrimination limitations. When a detector trained on one LLM is deployed and encounters a new LLM in production, near-original performance can be restored by collecting a small labeled sample of a few hundred emails and retuning the threshold, without retraining. This point is revisited in Section 5.

Two patterns within the matrix deserve note. First, GPT-4.1 and DeepSeek 3.2 transfer well to each other even at the default threshold (0.993 and 0.982), suggesting their stylometric signatures occupy adjacent regions of the feature space. Second, Llama 3.3 70B is the structural outlier: it is the most affected source, whether it is on the training or evaluation side. After recalibration, Llama continues to show the smallest F1 score (0.921–0.960), suggesting a small residual discriminative gap that recalibration cannot close.

### 4.5 Task C: cross-dataset human verification

To verify that the cross-model gap in Task B is driven by the LLM side rather than the human side of the data, we ran 20 cross-dataset human evaluations: training on humans from one source and evaluating on humans from a different source, with all LLMs. F1 scores ranged from 0.926 (when training on the Enron subset and evaluating on CEAS-08) to 0.999 (Nazario → CEAS-08), with a median of 0.978. The drop relative to intra-source training is below five percentage points in 19 of 20 cells. Human-side variation, therefore, contributes negligibly to the cross-model gap observed in Task B.

### 4.6 Task D: aggregated-pool detector

A single XGBoost detector trained on the union of all three LLMs against humans achieves an F1 score of 0.997 on each individual LLM and an AUC-ROC of 0.999 across all three cases. The aggregated detector essentially closes the cross-model gap under the default threshold, providing strong evidence in favor of multi-source training as the operational recommendation. This addresses RQ4: pooling phishing from multiple LLMs produces a robust universal detector.

### 4.7 Feature importance analysis

**Figure 4:**
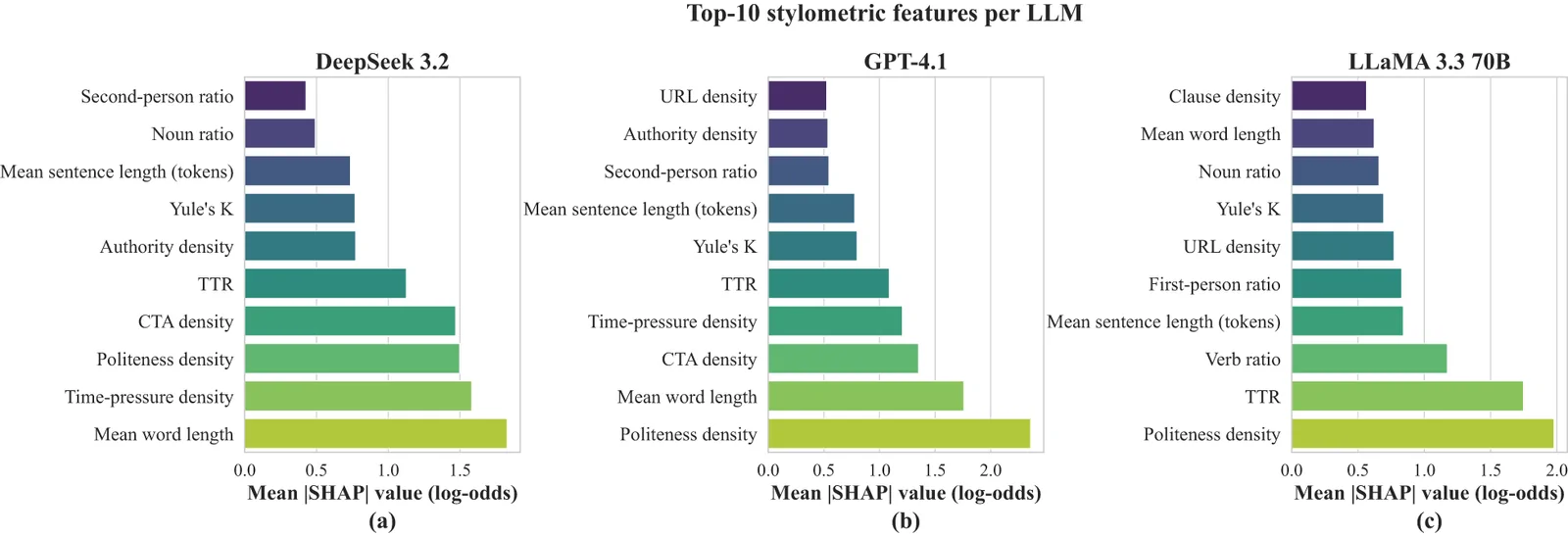
Top ten stylometric features ranked by mean absolute SHAP value for each LLM: **(a)** DeepSeek 3.2; **(b)** GPT-4.1; **(c)** Llama 3.3 70B.

**Figure 5:**
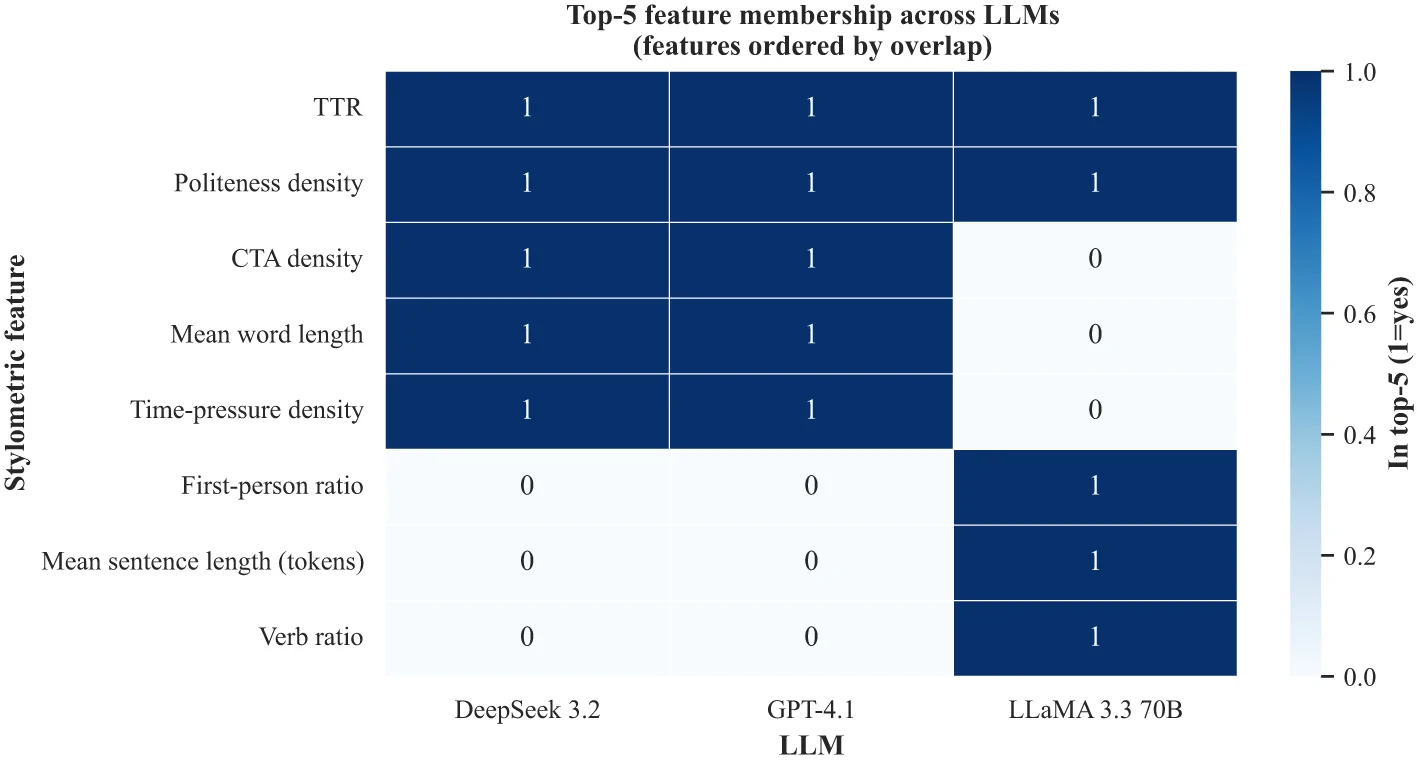
Top-5 feature membership across the three LLMs. A value of 1 indicates that the feature is in the top 5 most important features for that LLM.

SHAP analysis was conducted on each intra-model XGBoost classifier. Figure 4 presents the top ten features for each LLM, while Figure 5 displays the membership of each feature in the top five across the three models.

Two features, politeness density and type-token ratio, appear in the top five for all three models. These features constitute a stable subset of stylometric markers and provide the most robust foundation for cross-model detection. A second tier of features, including mean word length, call-to-action density, and time-pressure density, appears in the top five for two of the three models (GPT-4.1 and DeepSeek 3.2), but not for Llama. Three features are unique to Llama: verb ratio, first-person pronoun ratio, and mean sentence length in tokens. This distribution aligns with the cross-model matrix in Figure 3, where GPT-4.1 and DeepSeek 3.2 share most stylometric signatures, while Llama demonstrates a distinct profile.

This finding addresses RQ3: a stable subset of two features persists across all three modern LLMs evaluated, while model-specific signatures account for part of the residual gap observed for Llama in Section 4.4.

## 5 Discussion

### 5.1 Interpretation of findings

Three principal findings emerge. First, intra-model detection performance is consistently high across all three LLMs (F1 ≥ 0.95), thereby confirming and extending the results of Opara et al. (2025) to a broader set of generators, including the open-weight Llama 3.3 70B. Second, raw cross-model transferability under the default threshold is limited (gap = 28.0 percentage points); however, this gap primarily reflects a calibration artifact rather than a failure of discrimination, as the AUC-ROC remains above 0.96 in every off-diagonal cell. Third, two operational solutions are identified: threshold recalibration on a small target sample (reducing the gap to 4.0 percentage points) or aggregated-pool training, which effectively eliminates the gap and yields a universal detector with F1 = 0.997.

To the best of our knowledge, the threshold-calibration finding has not been previously documented in the stylometric phishing detection literature. This result reframes the cross-model challenge from one of fundamental incompatibility between LLM signatures to a matter of practical calibration. For security operations center (SOC) deployment, this distinction is significant: retraining a detector on phishing generated by a newly emerged LLM requires substantial labeling effort, whereas collecting a few hundred labeled samples for threshold tuning is feasible within a short timeframe.

The observed asymmetry in the transferability matrix is also noteworthy. Mutual transferability between GPT-4.1 and DeepSeek 3.2 remains high even at the default threshold, indicating that their stylometric outputs occupy similar regions of feature space. In contrast, Llama 3.3 70B serves as a structural outlier: detectors trained on this model do not generalize well, and detectors evaluated on it exhibit significant performance degradation. The feature-importance analysis in Section 4.7 elucidates this pattern: Llama's most salient features (verb ratio, first-person pronoun ratio, and sentence length) are largely distinct from those of the other two models, with only politeness density and type-token ratio (TTR) overlapping. This suggests that Llama's instruction-tuning and reinforcement learning from human feedback (RLHF) pipeline generates phishing prose with a more active register, characterized by higher verb density, greater use of personal voice, and shorter sentences, compared to the more nominal and formal registers of GPT-4.1 and DeepSeek 3.2. Whether this pattern generalizes to other open-weight models remains an empirical question for future research.

### 5.2 Comparison with prior work on AI-generated phishing detection

Table 7 situates our results against the directly relevant literature on AI-generated phishing detection. The comparison yields insights in three key areas.

**Table 7:** Quantitative comparison with directly relevant prior work on AI-generated phishing detection.

Study	Approach	Generators used	Best performance	Cross-model	Calibration
Opara et al. (2025)	Stylometric (47 feats) + XGBoost	1 (GPT-4o)	96.0% acc.	—	—
Koide et al. (2026)	LLM-as-detector	1 (GPT-4)	99.70% acc.	—	—
Chataut et al. (2024)	LLM-as-detector	2 (GPT-3.5, GPT-4)	GPT-4 best	—	—
This study	Stylometric (17 feats) + XGBoost	3 (GPT-4.1, DeepSeek 3.2, Llama 3.3 70B)	F1 = 0.95 to 0.97 (intra CV); 0.957 (cross + calib.)	✓	✓

*Generators* = number of distinct LLMs used to produce the phishing corpus. *Cross-model* = transferability across generators is evaluated. *Calibration* = the threshold-calibration component of the gap is isolated.

First, the intra-model performance we obtain (F1 ≥ 0.95 under cross-validation) is consistent with the headline results of prior work using a single generator: 96% accuracy in Opara et al. (2025) and 99.70% in Koide et al. (2026). This study replicates, across three distinct generators (including the open-weight Llama 3.3 70B), a finding previously established only within a single LLM. This replication constitutes a contribution by demonstrating that intra-generator detectability of LLM phishing is not exclusive to GPT-derived corpora.

Second, no prior study in this line evaluates cross-model transferability. In every cited work, the same generator (or LLM family) is used to produce the phishing corpus and to evaluate the detector. Our 3 × 3 transferability matrix is, to our knowledge, the first quantitative evaluation of how stylometric phishing detectors behave when the source LLM changes between training and deployment. The documented 28.0 percentage-point default-threshold gap represents a novel finding that prior work could not have identified due to their experimental design.

Third, no prior study decomposes the transferability gap into a discriminative component (whether the classifier still separates the classes) and a calibration component (whether the decision threshold remains valid). Our result that AUC remains above 0.96 in every off-diagonal cell, while the default-threshold F1 collapses, reframes the cross-model problem as one of practical calibration rather than a fundamental incompatibility. The 86% closure of the gap by threshold recalibration on a small target slice is, to our knowledge, the first such decomposition reported for phishing detection. Notably, the high AUC observed under cross-model shift contrasts with the more pessimistic outcomes reported for neural LLM-text detectors, where both AUC and threshold-dependent metrics often degrade simultaneously. This contrast indicates that hand-engineered stylometric features may offer greater transferability than learned representations, albeit with a modest limitation in maximum achievable accuracy.

In contrast to the offensive line of research (Roy et al., 2024; Heiding et al., 2024, 2026), which demonstrates that LLMs can generate phishing emails of human-expert quality at significantly reduced cost, the present results complement this evidence by showing that defensive systems do not require complete retraining when a new generator emerges. Instead, a small set of labeled samples is sufficient to restore detection performance to near-original levels.

### 5.3 Practical implications for security operations centers

Based on these results, three operational recommendations are proposed:

- Stylometric detectors intended for production deployment should be evaluated under cross-model conditions prior to operational reliance, even if intra-model F1 scores are near-perfect. Default thresholds are unlikely to transfer effectively.
- Upon the introduction of a new LLM in production traffic, recalibrate the detection threshold using a small labeled validation set (comprising a few hundred samples) prior to retraining. This approach restores near-intra-model performance with minimal additional effort.
- If labeling is feasible, training on the combined phishing data from multiple LLMs is recommended. An aggregated-pool detector achieved F1 = 0.997 across all three models in these experiments, providing a robust baseline for deployment.

Regarding operational scope, the detector evaluated in this study distinguishes human-written from LLM-generated phishing, rather than differentiating phishing from legitimate email. It is intended to serve as a single signal within a defense-in-depth pipeline. This signal should be combined with conventional layers, including URL reputation, header analysis, and attachment scanning. In this configuration, the cost of misclassification does not impact legitimate senders.

Beyond these specific recommendations, a broader implication is that adversarial techniques are continually evolving. The models currently used to generate phishing content will likely be replaced within months. Therefore, detectors should be designed and evaluated with the expectation of distributional drift, and recalibration may serve as a more practical first-line response than full retraining.

### 5.4 Limitations

Several limitations should be acknowledged; however, none undermine the study's central findings. First, only three large language models (LLMs) were evaluated. These models were intentionally selected to represent distinct training paradigms: These models include a commercial frontier reinforcement learning from human feedback (RLHF) model (GPT-4.1), an open-weight instruction-tuned model (Llama 3.3 70B), and an efficiency-optimized alternative (DeepSeek 3.2). The observed qualitative pattern, high intra-model F1, a substantial default-threshold gap, and a minimal recalibrated gap, is unlikely to change direction with the inclusion of additional models such as Mistral Large or Gemini. However, confirming the magnitude of these effects on further generators is a logical extension. Second, the study is limited to English. While English predominates in the phishing literature and accounts for the largest proportion of real-world targets, cross-lingual transferability remains a significant open question, particularly because stylometric features may behave differently across typologically distant languages. Third, the corpus represents a snapshot of LLM behavior at the time of generation. As API providers continuously update their models, absolute performance metrics may shift with new releases. This risk is mitigated by releasing the generation prompts, model identifiers, and generation parameters, which enable future replications to monitor drift over time. Fourth, the study focuses exclusively on email body text and does not incorporate header, URL, or attachment signals that operational filters typically use in combination. Therefore, the reported results should be interpreted as a lower bound on the performance of an integrated detection system; the stylometric signal assessed here is intended as one component within a defense-in-depth pipeline rather than a standalone solution. Fifth, the prompting strategy for generating the LLM corpus employs a single roleplay template. The prompt was fixed to control for a key confound in cross-model comparison: differences attributable to the model rather than the prompt. Alternative strategies, such as chain-of-thought, persona-based, or iterative humanization, may yield stylometrically distinct outputs and warrant dedicated follow-up studies. Sixth, the threshold-recalibration analysis assumes the availability of a small labeled sample from the target LLM. This assumption is realistic in most operational contexts, where analysts review a subset of incoming mail and generate labels as part of routine triage. Fully zero-shot deployment, in which no labeled target samples are available, represents a more stringent scenario for which unsupervised calibration methods are a promising area for future research.

## 6 Conclusion

This study conducted a cross-model evaluation of phishing detectors targeting emails generated by large language models (LLMs). A balanced dataset of 9,986 emails was assembled, comprising human phishing emails from five public sources (selected from an initial pool of six, with lingspam excluded due to length-distribution considerations) and phishing emails generated by GPT-4.1, DeepSeek 3.2, and Llama 3.3 70B. Seventeen stylometric features were extracted, and two classifier families were assessed under five experimental conditions: intra-model, cross-model, threshold-recalibrated, cross-dataset, and aggregated-pool settings.

The primary empirical finding is that the cross-model transferability gap observed in stylometric phishing detectors, although substantial under the default decision threshold (28.0 percentage points in F1), is mainly attributable to threshold calibration rather than a loss of discriminative capability. The receiver operating characteristic curve area remained above 0.96 in all off-diagonal cases. Threshold recalibration using a 30% subset of the target generator reduced the gap to 4.0 percentage points, representing an 86% reduction. An aggregated-pool detector trained on the combined data from all three LLMs achieved an F1 score of 0.997 on each individual generator. Politeness density and type-token ratio consistently ranked among the top five most important stylometric features across all three LLMs, providing a stable foundation for cross-model detection. In contrast, a distinct Llama-specific signature, comprising verb ratio, first-person pronoun ratio, and sentence length, explains the residual gap associated with that generator.

In summary, this paper contributes a balanced multi-LLM phishing corpus for community use, a systematic cross-model evaluation, and insights into the discriminative and calibration components. It also identifies stable and model-specific stylometric features and provides operational recommendations for security operations center deployment, including threshold recalibration as a cost-effective adaptation.

### 6.1 Emerging trends and future directions

Several emerging trends are expected to influence the next stage of research on AI-generated phishing detection.

The first trend is the rapid diversification of the generator landscape. As frontier reasoning models, multimodal large language models (LLMs), and open-weight model families continue to proliferate, the assumption that a detector can be trained once and deployed indefinitely is no longer tenable. Future research should evaluate stylometric and neural detectors against a broader and continuously updated panel of generators, including Mistral Large, Gemini, frontier reasoning models, and locally fine-tuned LLMs accessible to adversaries. A logical extension of the present study is the development of a continuously maintained transferability benchmark in which generator panels are updated quarterly.

The second trend is the transition from text-only to multimodal and multi-turn phishing. Voice cloning, deepfake video, and agentic phishing campaigns that adapt content in response to victim behavior represent emerging threats that extend beyond static email detection. The stylometric framework developed in this study serves as a baseline defense against a limited subset of this threat landscape. Integrating stylometric signals with multimodal and behavioral cues constitutes a priority research direction.

The third trend involves unsupervised and self-supervised calibration. The threshold-recalibration approach presented in this paper assumes the availability of a small labeled sample from the target generator. However, in practical applications, fully zero-shot deployment scenarios are common. Therefore, methods that calibrate decision thresholds without target labels, such as distribution-matching, prior shift adaptation, or self-training, represent an important area for future research.

The fourth trend is adversarial robustness against register shifting. Our error analysis showed that LLM phishing produced in a bureaucratic register evades the stylometric detector. Iterative paraphrasing, adversarial prompting, and persona-swapping techniques are expected to push generated phishing content further across the decision boundary. Future work should evaluate stylometric detectors under such adversarial transformations and develop defenses (e.g., adversarial training, register-aware features) to mitigate these risks.

The fifth trend is the integration of these methods with operational phishing-detection pipelines. The authorship signal evaluated in this study represents one component of a defense-in-depth strategy. Integrating this signal with URL reputation, header analysis, attachment scanning, and behavioral signals into a unified production filter is a logical next step for operational deployment. This includes the design of cost-aware threshold policies that account for the asymmetric consequences of false positives and false negatives in real-world traffic.

The broader implication is that defensive techniques must be designed under the assumption that the offensive side is not stationary. The same models that produce phishing today will be superseded within months. Stylometric detection, threshold recalibration, and aggregated-pool training together offer a practical strategy for maintaining up-to-date defenses at a fraction of the cost of full retraining. The released corpus and code are intended to support further research in this area.

## Data Availability

The datasets presented in this study can be found in online repositories. The names of the repository/repositories and accession number(s) can be found below: https://doi.org/10.5281/zenodo.20250116.
